# DAB Signal Preprocessing for Passive Coherent Location

**DOI:** 10.3390/s22010378

**Published:** 2022-01-05

**Authors:** Gustaw Mazurek

**Affiliations:** Faculty of Electronics and Information Technology, Warsaw University of Technology, Nowowiejska 15/19 Street, 00-665 Warsaw, Poland; g.mazurek@elka.pw.edu.pl

**Keywords:** passive bistatic radar, passive coherent location, digital audio broadcasting, DAB illuminated PCL, signal preprocessing

## Abstract

Digital Audio Broadcast (DAB) transmitters can be successfully used as illumination sources in Passive Coherent Location (PCL). However, extending the integration time in such a configuration leads to the occurrence of periodical artifacts in the bistatic range/Doppler plots, resulting from the time structure of the DAB signal. In this paper, we propose some methods of signal preprocessing (based on symbol removal, substitution by noise, and duplication) that operate on the DAB transmission frame level and improve the received signal’s correlation properties. We also demonstrate that two of these methods allow us to avoid the mentioned artifacts and thus to improve the quality of the range/Doppler plots with detection results. We evaluate the performance of the proposed methods using real DAB signals acquired in an experimental PCL platform. We also provide the analysis of the Signal to Noise Ratio (SNR) during the detection of a moving target which shows that the proposed solution, based on symbol duplication, can offer around 3 dB of gain in SNR. Finally, we carry out the computational complexity analysis showing that the proposed method can be implemented with a minimal cost after some optimizations.

## 1. Introduction

Broadcast radio and television transmitters, e.g., Frequency Modulated (FM), Digital Audio Broadcast (DAB), and Digital Video Broadcasting-Terrestrial (DVB-T) are commonly used as illumination sources for Passive Coherent Location (PCL) [1,2,3] due to their excellent availability, high level of emitted power, and significant area coverage. Moreover, the broad (and time-invariant) frequency spectrum of digital broadcast signals (DAB, DVB-T) has a positive impact on the performance of PCL detection [4,5] in terms of range, Doppler resolution [6], and predictable characteristics, when compared to analog FM transmission [7,8].

**Figure 1:**
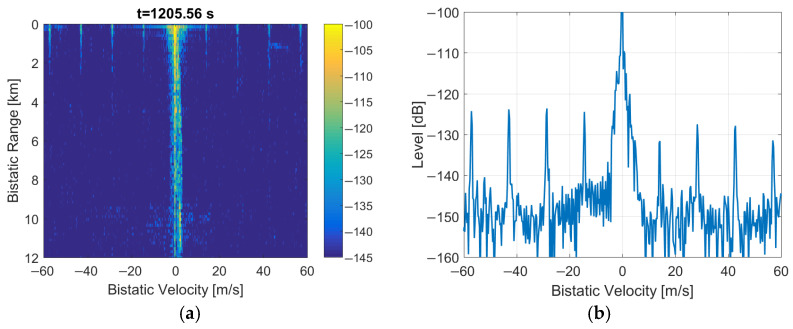
Bistatic velocity/range plot with artifacts (**a**) and its cross-section for *R* = 450 m (**b**).

The performance calculations and initial trials of DAB-illuminated PCL are presented in [4,9,10]. The results of experiments with DAB signals acquired during the NATO campaign APART-GAS 2019 (Active Passive Radar Trials Ground-based, Airborne, Sea-borne) in Poland [11] showed encouraging results in detecting airplanes of different sizes flying in the range up to 20 km [12] from the moving PCL platform [13]. However, due to the time-domain properties of the DAB signal, there were periodical artifacts sometimes visible in bistatic velocity/range plots that obscure the images with detection results. Examples of such images are shown in [12] and in Figure 1 as a reference. These disturbances appeared with an integration time extended to 5 s. They were repeated on the bistatic velocity axis with a period $v_{a}$ ≈ 14.3 m/s that is strictly related to the signal’s wavelength (λ = 1.37 m for the received DAB center frequency: 218.64 MHz) and the DAB transmission frame duration [14,15] ($T_{F}$ = 96 ms)

(1) $$v_{a}=\frac{\lambda }{T_{F}}.$$

Due to this connection with DAB signal timing, it is necessary to investigate the DAB signal structure to find its influence on the quality of PCL detection images.

Several signal processing methods are proposed in the literature to reduce the ambiguity peaks in the Orthogonal Frequency Division Multiplex (OFDM) signal (mainly DVB-T) for passive radar applications. However, it must be noted that the DAB signal, despite the same modulation scheme (OFDM), has a different structure [16] due to its synchronous design.

In [17], methods for reducing the ambiguity peaks in the cross-ambiguity function were investigated for the PCL system with DVB-T illumination signals. Three methods were proposed to replace the elements responsible for the ambiguity peaks with ideal 64-Quadrature Amplitude Modulation (QAM) symbols, zero power components, and finally, random segments. The results show a significant reduction of the ambiguity peaks but with some undesired reduction in detection capabilities. Since DVB-T signal is assumed, whose structure differs from DAB signal [16], some effects resulting from the DAB signal structure (i.e., repeatable symbols at the beginning of each frame and interrupted RF transmission) were not considered. In this paper, we focus mainly on these DAB-specific issues.

As demonstrated in [18], the side peaks of the ambiguity function of OFDM signals are caused by the periodicity of pilots. The solution was proposed to eliminate the side peaks in the received signals to improve the performance of cognitive radio-based PCL. A theoretical analysis was provided for general OFDM signals (e.g., WiFi, WiMax, DVB-T) and confirmed by a series of simulations, but no experimental results with the real signals were presented. In [7], a multistage processing algorithm was developed for disturbance cancellation and target detection based on projections of the received signal in a subspace orthogonal to both the disturbance and previously targets. The described work focused on the use of FM radio signals, and experimental verification was performed only with FM-illuminated PCL. 

An efficient architecture of a coherent integration processor for DVB-T-based passive bistatic radar was proposed in [19]. The proposed architecture consists of a frequency-domain pulse compression module and a decimated keystone transform module based on the chirp *z*-transform to compensate for the range migration effect caused by high-speed targets. The effectiveness and validity of the proposed design have been verified through real-data experiments. A modular and reconfigurable passive radar architecture was also shown in [20] based on a software-defined approach. Thanks to efficient implementation in a multi-core machine, the system was able to perform real-time processing of one or two surveillance channels and one reference channel. The described signal processing pipeline consisted of beamforming, adaptive filtering, cross-ambiguity function calculation, adaptive detection, and plot extraction. In [21], a signal pre-processing method based on a generalized multi-channel adaptive filter was developed to suppress the sea clutter in a passive radar. The effectiveness of the proposed approach was verified using real-data results with the illumination from a digital television broadcast transmitter. A multi-stage procedure designed to remove clutter and strong echoes from moving targets was shown in [22]. The proposed solution was intended to improve the dynamic range in PCL and noise radars and thus facilitate the detection of weak targets. However, it was verified using only simulations. A space-time Constant Modulus Algorithm (CMA) for multipath removal on the reference signal in PCL was proposed in [23]. The adaptive equalization of the reference signal based on an array of antennas and multiple receiving channels was shown in simulations to recover the performance loss caused by multipath contribution.

In [24], the reconstruction of the reference OFDM signal by demodulation and remodulation was presented to produce the perfect copy of the transmitted signal and thus to remove the influence of multipath propagation. Additionally, digital signal equalization was proposed to remove the spurious peaks in the ambiguity function caused by the presence of pilot sequences. The DVB-T signal’s cross-ambiguity function was also analyzed, and several methods of its improvement for passive radar were proposed in [6,25,26,27,28,29]. A preprocessing technique designed to improve the range resolution of PCL and to reduce the unwanted side peaks in the ambiguity function by exploiting multiple signals (i.e., several broadcast channels) was proposed in [6,30]. However, since the DVB-T signal was considered, the effects resulting from the specific structure of the DAB signal were not taken into account, and the obtained results cannot be directly translated to DAB-illuminated PCL.

A target detection method by correlating a surveillance signal with a reconstructed reference signal in DAB-based PCL was introduced and validated in [8]. It was experimentally confirmed that reference signal reconstruction could be effectively employed for direct signal interference suppression and PCL detection algorithms; however, the integration time was only set to 500 ms, which limits the Doppler resolution.

The preprocessing method for DAB signal in PCL application was described in [31]. However, the presented results were limited to only eliminating the periodic ambiguity peaks in the signal’s autocorrelation function, with no investigation into the influence on the PCL detection results.

In this paper, we continue the work presented in [31]. We begin with a short review of the essential properties of the DAB signal. Then we present four preprocessing methods that improve the correlation properties of the DAB signal. Finally, we investigate their influence on the quality of bistatic velocity/range plots with PCL detection results obtained from real signals acquired in the field. We show that two of the presented methods eliminate the periodical artifacts from the bistatic velocity/range plots reported in [12]. The main contribution of this work is a novel, simple yet effective algorithm of received signal preprocessing in the temporal domain (at the DAB frame level) that improves the readability of bistatic velocity/range images obtained from DAB-illuminated PCL in cases of extended integration time (i.e., much longer than 96 ms).

## 2. Materials and Methods

### 2.1. Signal Acquisition and PCL Processing

For the experiments described in this paper, DAB signals acquired during APART-GAS 2019 campaign [11,12] have been employed. The illuminating transmitter was localized near Koszalin, Poland, and operated at a VHF frequency of 216.84 MHz with an Effective Radiated Power (ERP) level of 3.2 kW and vertical polarization. The PCL signal acquisition platform was moving [13] because of the installation on a ship sailing on the Baltic Sea, near the Polish coastline. The PCL receiver was based on a lightweight, four-channel configuration with RSP duo devices described in [32]. Further details of the system setup, antenna array, and supplementing hardware have been described in [12]. Two baseband DAB signals acquired coherently from directional antennas (the reference and the surveillance) have been digitized with 14-bit ADCs (sample rate *F*_s_ = 2 MHz) and stored. The remaining two signals were reserved for multi-channel processing planned in the further research. 

The signal processing pipeline used for obtaining the bistatic velocity/range plots with PCL detection results consists of the clutter-removal filter (based on an adaptive lattice filter) to suppress the direct path interference in the surveillance channel and the cross-ambiguity function calculator, as described in [12].

### 2.2. DAB Signal Structure

There have been four modes of DAB transmission defined historically [14], but in practice, only Mode I has remained in the current specification (ver. 2.1.1) since January 2017 [15]. The transmitted signal is divided into frames and symbols in the time domain and subcarriers in the frequency domain. The parameters of the DAB signal are summarized in Table 1.

**Figure 2:**
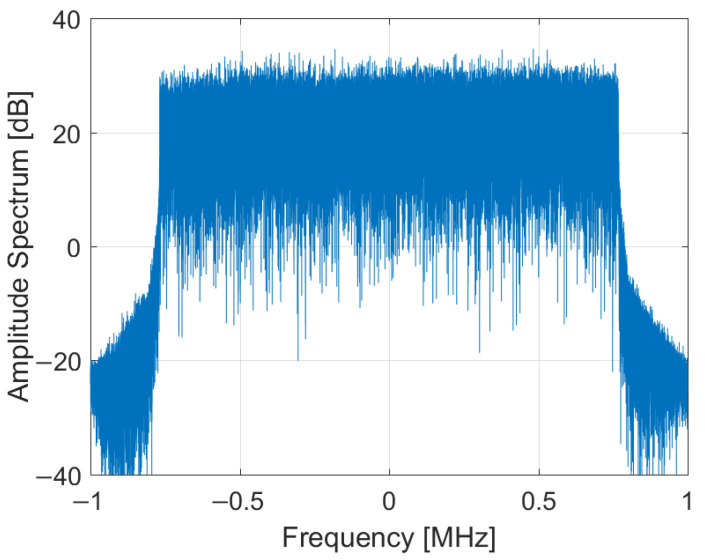
Frequency spectrum of the DAB signal.

The DAB transmission employs a Coded Orthogonal Frequency Division Multiplex (COFDM) modulation scheme with a signal bandwidth of 1.536 MHz [16]. The spectrum of the acquired DAB signal is shown in Figure 2.

**Figure 3:**
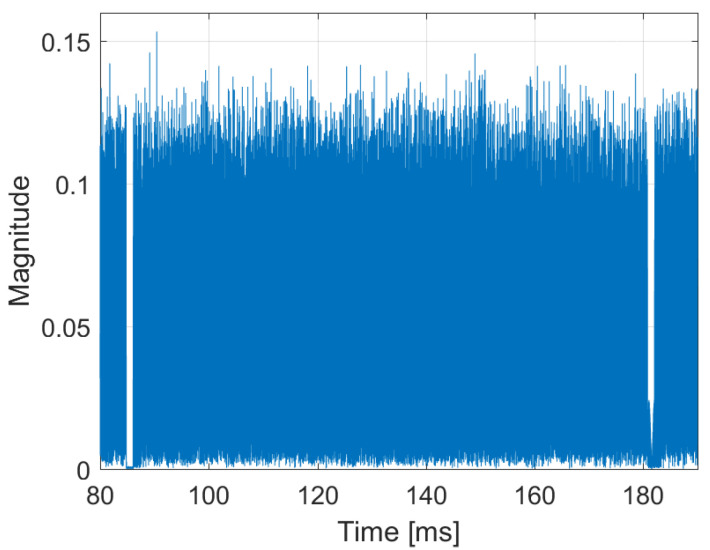
Amplitude plot of the DAB signal (one complete frame).

In the temporal domain, the signal consists of consecutive frames of constant duration ($T_{F}$). Each frame begins with the Null symbol, which is a period with RF emission turned off that allows the coarse synchronization of the receiver. The occurrence of the Null symbol is clearly visible in the signal’s envelope, as depicted in Figure 3.

**Figure 4:**
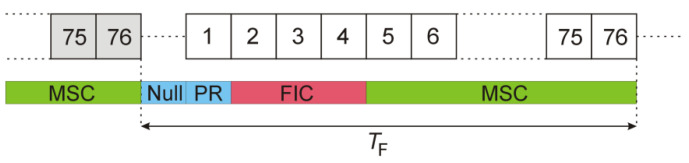
DAB frame organization.

The first symbol transmitted in the frame with the RF carrier turned on is a Phase Reference (PR) symbol. It is intended for the synchronization of the time, frequency, and phase of the following data symbols. Since it consists of OFDM subcarriers transmitted with strictly defined phase offsets, it is a pure deterministic part of the signal, repeated in every DAB frame. The structure of a complete DAB frame is illustrated in Figure 4. After the PR symbol, three Fast Information Channel (FIC) OFDM symbols are transmitted with a significantly reduced data rate for system organization and service control purposes [15] (p. 16). Eventually, the remaining 72 OFDM symbols of the Main Service Channel (MSC) are transmitted with the full data rate containing the compressed digital audio streams and, occasionally, additional data service components [15] (p. 11).

### 2.3. DAB Signal Correlation Properties

**Figure 5:**
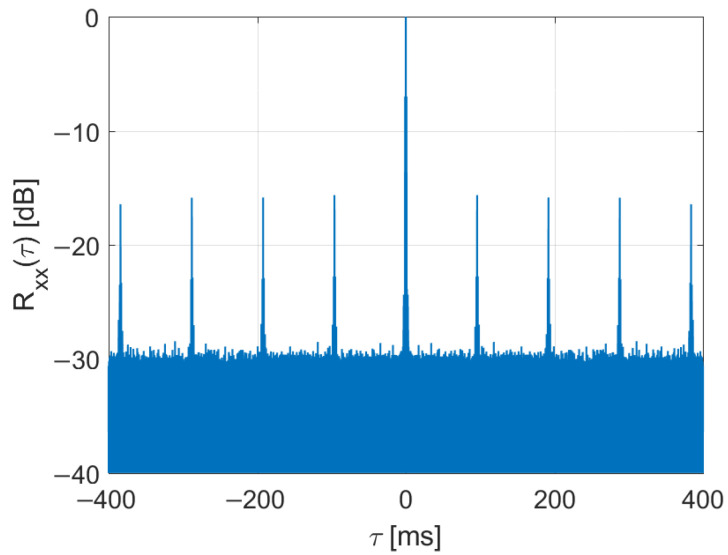
Autocorrelation of DAB signal.

The spectrum of the acquired DAB signal and its amplitude waveform have been depicted in Figure 2 and Figure 3, respectively. The autocorrelation function computed for this signal is shown in Figure 5, with the power levels normalized to the central correlation peak. 

Besides the peak at *τ* = 0, several side peaks at multiples of $T_{F}$ occur. Such ambiguity peaks of the illuminating signal are generally undesirable in radar applications [17,26,29]. As shown in [31], these side peaks come from the deterministic parts of the DAB signal, i.e., the PR and FIC symbols in the frame. Therefore, the removal or substitution of these symbols improves the signal’s autocorrelation function, as will be demonstrated in the following subsection.

### 2.4. DAB Signal Preprocessing

A method of DAB signal preprocessing was presented [31] that works in the time domain with the complex baseband DAB signal *x*(*n*). That method employs signal envelope measurements to detect the Null symbol, which occurs at the start of each frame. The envelope is smoothed by processing the amplitude of the x(*n*) signal in a moving average filter with *N* = 128 taps

(2) $$e\left(n\right)=\frac{1}{N}\overset{N-1}{\underset{k=0}{\sum }}\left|x\left(n-k\right)\right|.$$

The mean signal level is estimated in a similar way by long-term averaging (e.g., *M* = 10^6^) of the amplitude

(3) $$e_{\mathrm{avg}}\left(n\right)=\frac{1}{M}\overset{M-1}{\underset{k=0}{\sum }}\left|x\left(n-k\right)\right|.$$

If the signal envelope *e*(*n*) falls below the fixed threshold, i.e., when *e*(*n*) < 0.3 *e*_avg_(*n*), the beginning of the Null symbol is detected. When the signal envelope rises above the threshold in this state, the beginning of the PR symbol is detected, and the time instant of this situation ($n_{0}=n$) is stored as a reference for the subsequent operations. This method has been shown [31] to perform satisfactorily with the high-SNR signal, for example, when *x*(*n*) is the reference signal from the PCL receiver. However, if synchronization to noisy signals is required, more robust frame synchronization methods (e.g., based on the correlation techniques) may still be implemented [33,34,35].

#### 2.4.1. Clearing of PR and FIC Symbols

**Figure 6:**
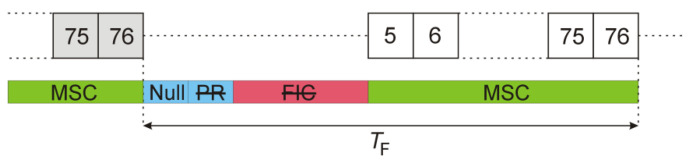
DAB frame with cleared PR and FIC symbols.

After detecting the PR, the part of the baseband signal containing the four initial symbols of the frame (PR, 3 × FIC) is cleared by changing the relevant samples to zero

(4) $$x\left(n\right)≔0,\;n=n_{0}-N_{margin},\ldots ,n_{0}+4\times N_{symb}$$

where $N_{\mathrm{margin}}=128$ is applied to compensate the envelope detector’s latency, and $N_{\mathrm{symb}}=2492$ is the OFDM symbol duration expressed in samples. The frame structure after this modification is shown in Figure 6. The deterministic components (PR and FIC symbols) that repeat from frame to frame have been removed, and only a random part (i.e., MSC symbols) has been left. 

**Figure 7:**
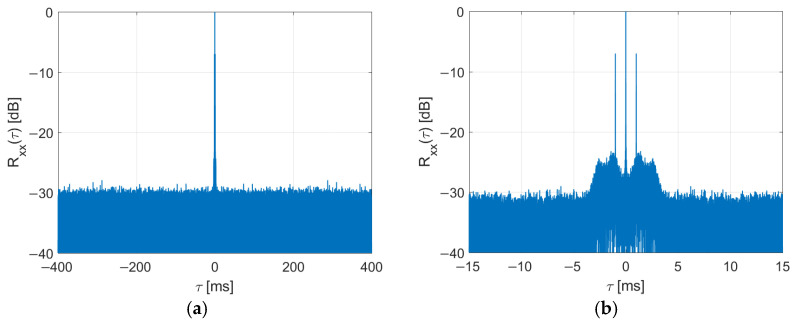
Autocorrelation function of DAB signal after clearing of PR and FIC symbols (**a**) and its close-up (**b**).

It has been shown [31] that after the PR and three FIC have been deleted from the received DAB frames, the periodical ambiguous peaks are no longer visible in the signal’s autocorrelation function, as depicted in Figure 7.

The side peaks at ±1 ms visible in Figure 7b result from the guard interval (and cyclic prefix insertion) in the OFDM signal. Several methods of reducing these peaks have already been investigated [17,25,26].

The same modification is performed synchronously (i.e., on the same sample indexes) on the signal $x_{s}\left(n\right)$ from the surveillance channel

(5) $$x_{s}\left(n\right)≔0,\;n=n_{0}-N_{\mathrm{margin}},\ldots ,n_{0}+4\times N_{\mathrm{symb}}$$

before feeding it to the input of the PCL detection algorithm. As a continuation of the work presented in [31], we investigate another three methods of DAB signal preprocessing for PCL applications, which are described further.

#### 2.4.2. Insertion of Synthetic Noise

**Figure 8:**
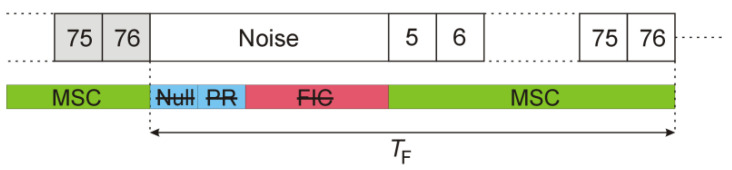
DAB frame structure with Null, PR, and FIC symbols substituted by noise.

As described previously, the time instant of the start of the frame ($n_{0}$) is determined with envelope detection. After that, the signal samples carrying the null, PR, and FIC symbols from both the reference and the surveillance channel are replaced by the complex noise

(6) $$x\left(n\right)≔w\left(n\right),\;x_{s}\left(n\right)≔w_{s}\left(n\right),\;n=n_{0}-N_{\mathrm{margin}}-N_{\mathrm{null}},\ldots ,n_{0}+4\times N_{\mathrm{symb}}$$

where w(n) and $w_{s}\left(n\right)$ are statistically independent realizations of the Gaussian noise (with their variances scaled to the estimated power level of the DAB signal), and $N_{\mathrm{null}}$ is the duration of the null symbol (in samples) that is slightly longer than in case of the data symbol ($N_{\mathrm{symb}}$) [14]. The frame structure after this modification is shown in Figure 8.

The received signals have constant power and no emission discontinuities, thanks to this modification. The periodical peaks are not visible in the signal’s autocorrelation function, as shown in Figure 7.

#### 2.4.3. Duplication of MSC Symbols in Place of Null, PR, FIC Symbols

After finding the start of the frame ($n_{0}$) as described previously, the signal samples carrying the null, PR, and FIC symbols from both the reference and the surveillance channel are replaced by the copies of the selected MSC symbols from the previous frame

(7) $$x\left(n\right)≔x\left(n-N_{s}\right),\;x_{s}\left(n\right)≔x_{s}\left(n-N_{s}\right),\;n=n_{0}-N_{\mathrm{margin}}-N_{\mathrm{null}},\ldots ,n_{0}+4\times N_{\mathrm{symb}}$$

where the time shift $N_{s}$ results from the index $N_{C0}$ ($5\leq N_{C0}\leq 72$) of the first MSC symbol that was copied from the previous frame into the position of the Null symbol

(8) $$N_{s}=\left(77-N_{C0}\right)\times N_{\mathrm{symb}}.$$

**Figure 9:**
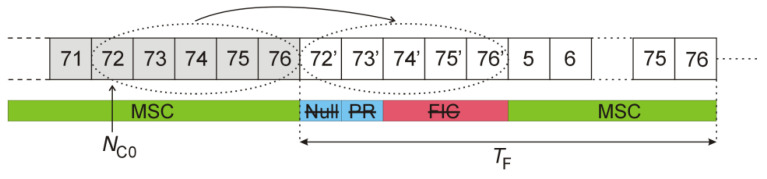
DAB frame structure with Null, PR, and FIC symbols replaced by MSC symbols.

The frame structure after this modification is shown in Figure 9.

**Figure 10:**
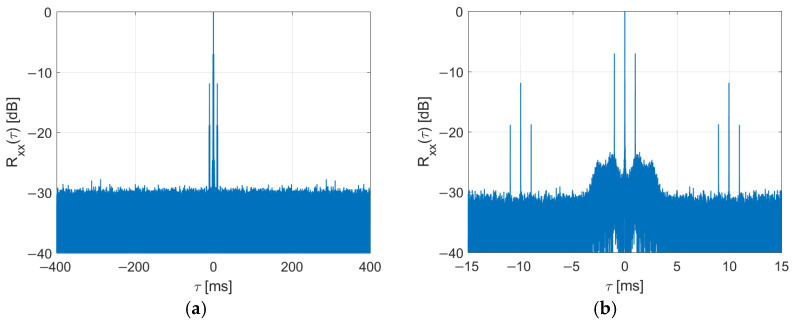
Autocorrelation of DAB signal after copying of MSC symbols (**a**) and its close-up (**b**).

The side effect of partial signal duplication (7) is the introduction of additional side peaks in the autocorrelation plot of the signal (with adjacent pairs of peaks resulting from the cyclic prefix insertion [17]), as depicted in Figure 10 for $N_{C0}=69$. However, the position of these peaks is strictly determined (8) and thus can be modified by selecting the copied symbols ($N_{C0}$).

#### 2.4.4. Duplication of MSC Symbol in Place of Null Symbol

**Figure 11:**
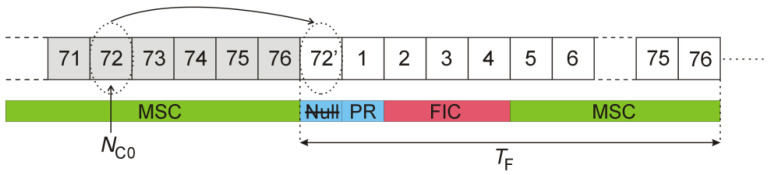
DAB frame structure with Null symbol replaced by MSC symbol.

After finding the start of the frame ($n_{0}$) as seen previously, the signal samples carrying the Null symbol from both the reference and the surveillance channel are replaced by copies of one MSC symbol selected from the previous frame

(9) $$x\left(n\right)≔x\left(n-N_{s}\right),\;x_{s}\left(n\right)≔x_{s}\left(n-N_{s}\right),\;n=n_{0}-N_{\mathrm{margin}}-N_{\mathrm{null}},\ldots ,n_{0}$$

where $N_{s}$, as previously (8), is determined by the index $N_{C0}$ ($5\leq N_{C0}\leq 76$) of the copied MSC symbol. The frame structure after this modification is shown in Figure 11.

**Figure 12:**
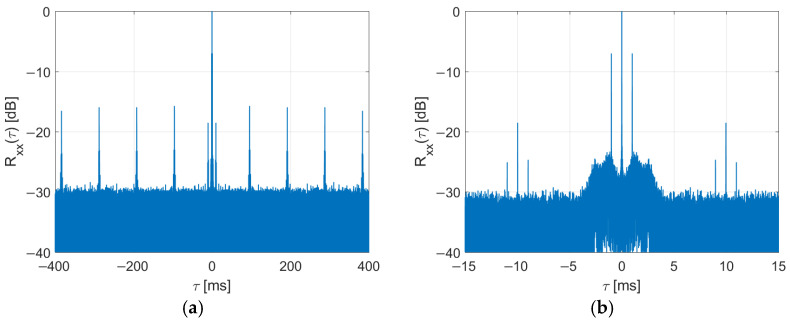
Autocorrelation of DAB signal with one MSC symbol copied in place of Null symbol (**a**) and its close-up (**b**).

As mentioned previously, additional side peaks have been introduced this way in the autocorrelation function of the signal. Additionally, because of unchanged PR and FIC symbols, the periodical side peaks in the autocorrelation function of the signal are still present, as shown in Figure 12 ($N_{C0}=69$). Besides these drawbacks, this kind of signal modification is interesting in PCL signal processing and will be further analyzed. It should be noted that the only modified part of the frame was the Null symbol with no RF signal emission. Since the Null symbol position is known, and PR, FIC symbols are left unchanged, the DAB frames after this modification can still be demodulated and decoded, for example, to reconstruct the reference signal [8,24].

## 3. Results

**Figure 13:**
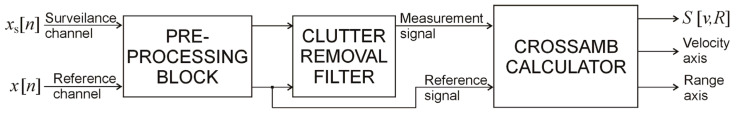
Signal processing pipeline used for performance evaluation.

The performance of the proposed methods of the DAB signal preprocessing has been compared with the help of the PCL signal processing algorithm described in [12], which consists of the clutter removal filter and the cross-ambiguity function calculator. The signal processing algorithms described in the previous section are implemented in the pre-processing block that is placed before the clutter removal filter (see Figure 13). The signal processing algorithms (implemented in Matlab environment) get the raw baseband signals acquired in the system described in [12] (with time delays and phase shifts corrected using the method based on pilot signals [32,36]) and return the cross-ambiguity function plots as output. The integration time was set to 5 s in all the following experiments. This allowed us to extend velocity resolution and thus to investigate the sea clutter structure (during the storm and calm seas) and distinguish slow-moving targets from the clutter, which, in general, is a challenging problem [21].

### 3.1. Clearing of PR and FIC Symbols

**Figure 14:**
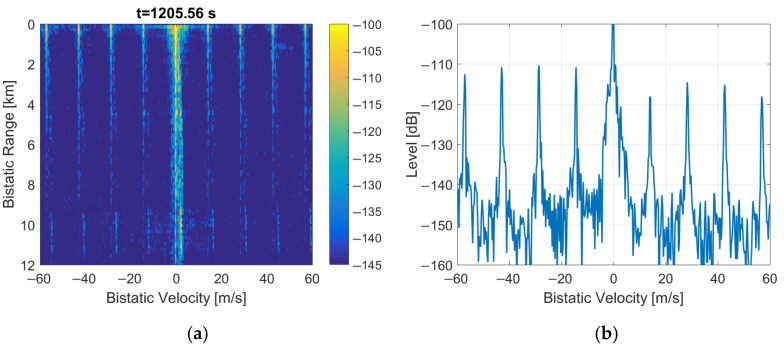
PCL detection results after clearing of PR and FIC symbols in the DAB frame: (**a**) bistatic velocity/range plane; (**b**) its cross-section at selected range.

**Figure 15:**
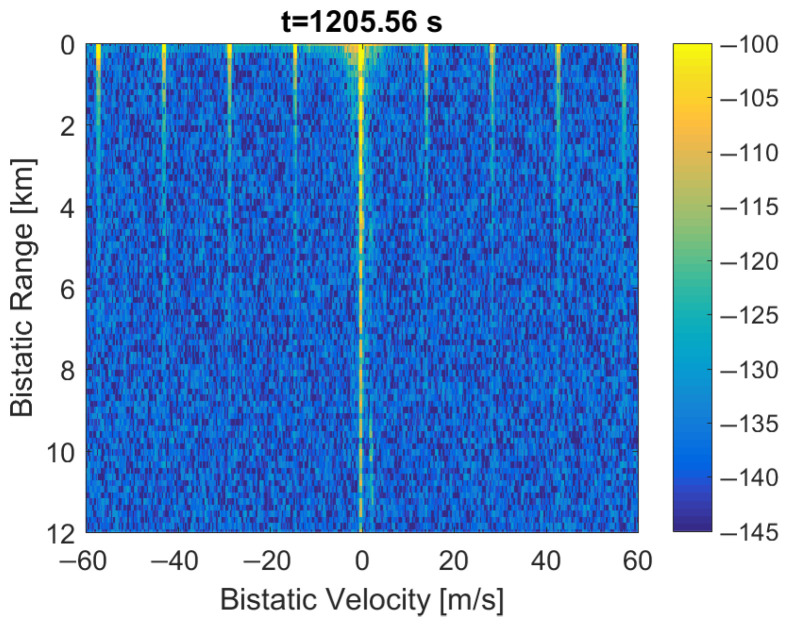
PCL detection results after the clearing of the PR and FIC symbols (bypassed clutter-removal filter).

After removing the periodical parts of the DAB frames (i.e., the PR and FIC symbols) by clearing their sample values, the periodical artifacts are still visible in the bistatic velocity/range (*v*/*R*) plane, as shown in Figure 14. What is essential, is that these artifacts are even intensified by ca. 15 dB when compared to Figure 1. One moving object is visible at *R* = 1.2 km, *v* = 50 m/s, but its echo will be obscured by the artifacts at bistatic velocities being multiplies of $v_{a}$ (1). This effect can cause false detections, and, in general, it degrades the detection and tracking the performance of the considered passive radar. The artifacts stay visible when the clutter-removal filter is bypassed (see Figure 15).

### 3.2. Replacing of Null, PR and FIC Symbols by Noise

**Figure 16:**
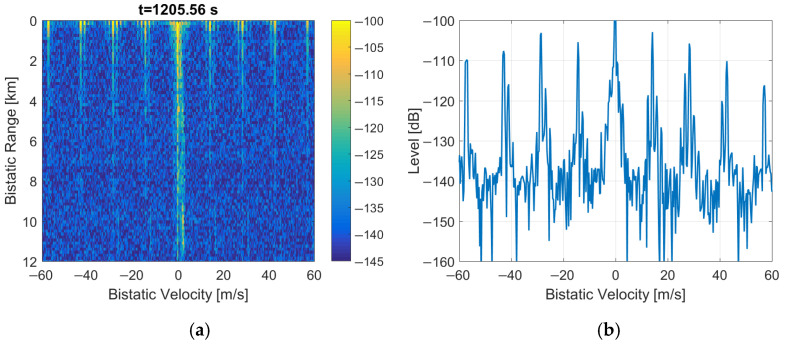
PCL detection results after noise insertion in place of Null, PR, and FIC symbols in the DAB frame: (**a**) bistatic velocity/range plane; (**b**) its cross-section at *R* = 450 m.

The replacement of the Null, PR, and FIC symbols by the noise leads to a raised noise floor in the detection results (as shown in Figure 16) since the introduced noise cannot be suppressed with the clutter-removal filter. The bistatic *v*/*R* plane looks similar to the previous case from Figure 15 with the bypassed clutter-removal filter, but the periodical artifacts are even more intensified. These artifacts are approx. 20 dB more intensive than in the reference (Figure 1). The moving object at *R* = 1.2 km is obscured by the noise and is therefore undetectable. 

**Figure 17:**
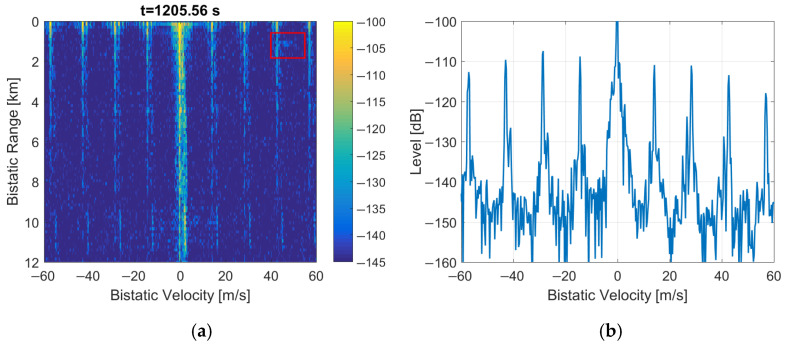
PCL detection results after the insertion of noise with reduced power: (**a**) bistatic velocity/range plane; (**b**) its cross-section at *R* = 450 m.

After reducing the power of the inserted noise by 6 dB, the noise floor is lower and the moving object at *R* = 1.2 km emerges from the noise, as marked in Figure 17 with a red rectangle. Further reduction of the inserted noise power results in an even lower noise floor. When the inserted noise power is at least 9 dB lower than the DAB signal power, the situation converges toward the case of clearing the samples of initial frame symbols, shown in Figure 14. However, it should be emphasized that with this method, the periodical artifacts are constantly visible in the bistatic *v*/*R* plane.

### 3.3. Duplication of Five MSC Symbols

**Figure 18:**
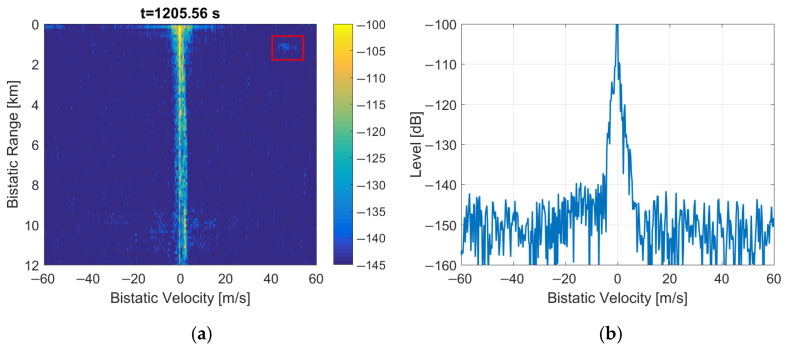
PCL detection results after MSC symbols duplication to null, PR, and FIC symbols: (**a**) bistatic velocity/range plane; (**b**) its cross-section at *R* = 450 m.

When the Null, PR, and FIC symbols at the beginning of the frame are replaced by the copies of selected five MSC symbols from the previous frame, the periodical artifacts are eliminated from the bistatic *v*/*R* plane as depicted in Figure 18 (their intensity is at least 20 dB lower than in Figure 1). After such a signal modification, the adaptive clutter-removal filter [12] properly removes the direct path interference, which yields a low background noise level visible in the plot. The echo from the moving object is visible on the bistatic *v*/*R* plane at *R* = 1.2 km (marked with a red rectangle). The characteristic spread spectrum of the sea clutter [21] can also be observed.

**Figure 19:**
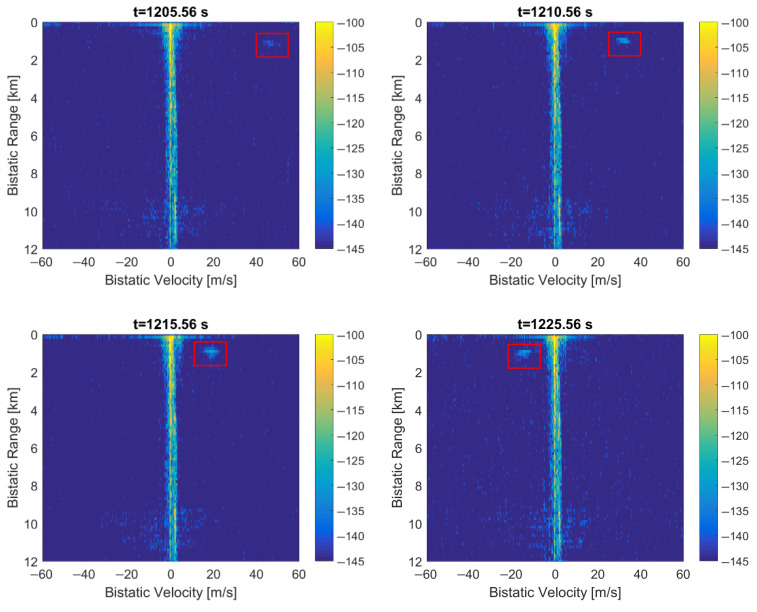

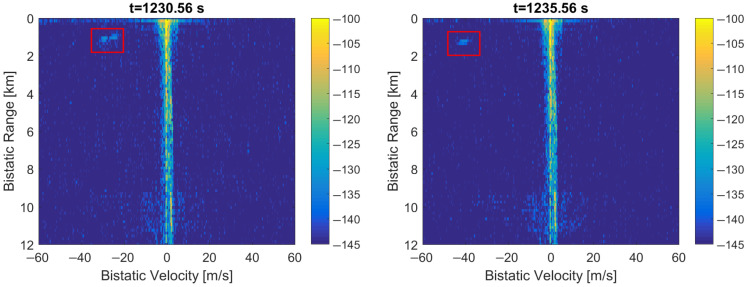
PCL detection results after MSC symbols duplication, subsequent time stamps.

The detection results of the considered moving object are shown in Figure 19 for the subsequent time stamps. For *t* = 1220.56 s, the echo from this object is at *R* = 900 m, *v* = 6.6 m/s, and it merges with the clutter strip at this scale (not shown here). Due to the long integration time in relation to the moving object’s velocity, migration of the detection results is observed. This effect may be compensated using more advanced coherent integration algorithms [19].

### 3.4. Substitution of the Null Symbol by Selected MSC Symbol 

**Figure 20:**
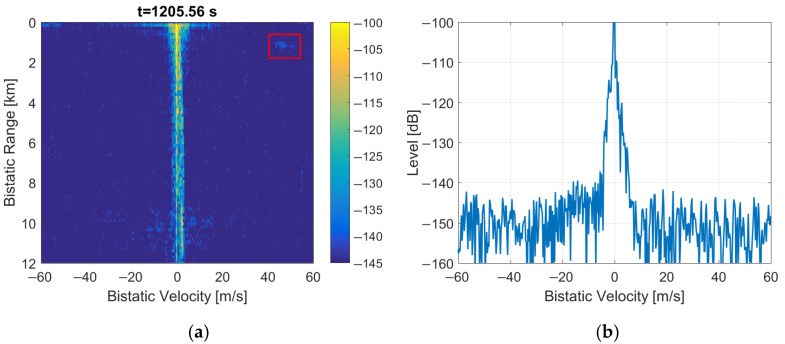
PCL detection results after MSC symbol duplication in place of Null symbol: (**a**) bistatic velocity/range plane; (**b**) its cross-section at *R* = 450 m.

The simplified method of copying just one MSC symbol from the previous frame in place of the Null symbol gives the same improvement (depicted in Figure 20) as in the previous case. The periodical artifacts are removed from the bistatic *v*/*R* plane, and the echo from the moving object is still visible at the low-level background noise.

## 4. Discussion

The PR and FIC symbols in the DAB frame constitute a deterministic part of the signal that results in periodic peaks in the autocorrelation function of the received signal. Another source of the ambiguity peaks is the presence of guard intervals, filled with the cyclic prefix, which is common in all systems with OFDM modulation. These unwanted peaks in the autocorrelation function can be removed by clearing certain parts of the received signal or filling them with noise. However, they correspond to relatively long distances in the range domain (150 km for the symbol duration, and 14,400 km for the frame duration $T_{F}$) [17] that lie far beyond the operating range of the considered passive radar [12] and thus have no visible influence on the plots with detection results.

**Figure 21:**
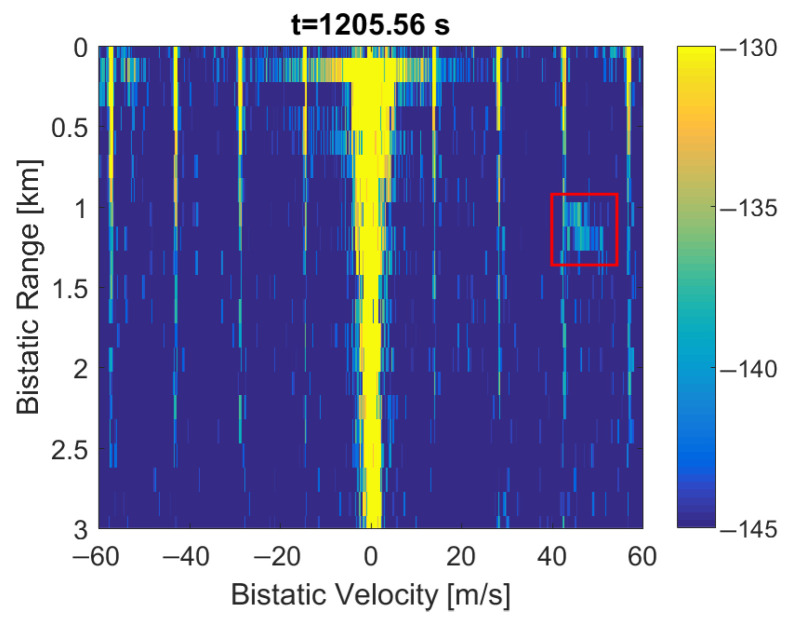
Bistatic velocity/range plot with artifacts and weak target (close-up).

The main problem of the considered system was the presence of periodical artifacts in the bistatic velocity/range plane with a long integration time (5 s) applied to extend resolution in the bistatic velocity. Such artifacts can cause false detections, obscure echoes from objects moving at specific velocities, and disturb tracking. In some situations, these artifacts can even be more intensive than the weak signals from the targets, as shown in Figure 1 and Figure 21, with different scales and target echo marked by a rectangle.

The experimental results described in this paper show that clearing PR and FIC symbols in the DAB frame (see Figure 6), despite correcting the autocorrelation function, does not remove the mentioned periodical artifacts, but quite the opposite, it makes them even more intensive. The second method (replacing the Null, PR, and FIC symbols with a noise) not only does not remove the artifacts but also raises the noise floor in the bistatic velocity/range plane, which makes the detection of objects difficult, or even impossible, as demonstrated in Figure 16 and Figure 17.

Finally, the periodical artifacts in the bistatic velocity/range plane could be removed by copying part of the previous DAB frame (i.e., the selected MSC symbols) in place of the Null, PR, and FIC symbols in the current frame (see Figure 9), synchronously in both the reference and the surveillance signals. Although an additional peak in the autocorrelation function is introduced in this way (see Figure 10), its position can be shifted beyond the operating range of the PCL radar by an appropriate selection of the copied MSC symbols (8). The example plots with moving object’s detection results after such a modification in the received signal are shown in Figure 18 and Figure 19, and in animations S2, S4 provided in the Supplementary Materials. The animations S1 and S3 are available as a reference and show the initial detection results without modifications in the received signals. 

**Figure 22:**
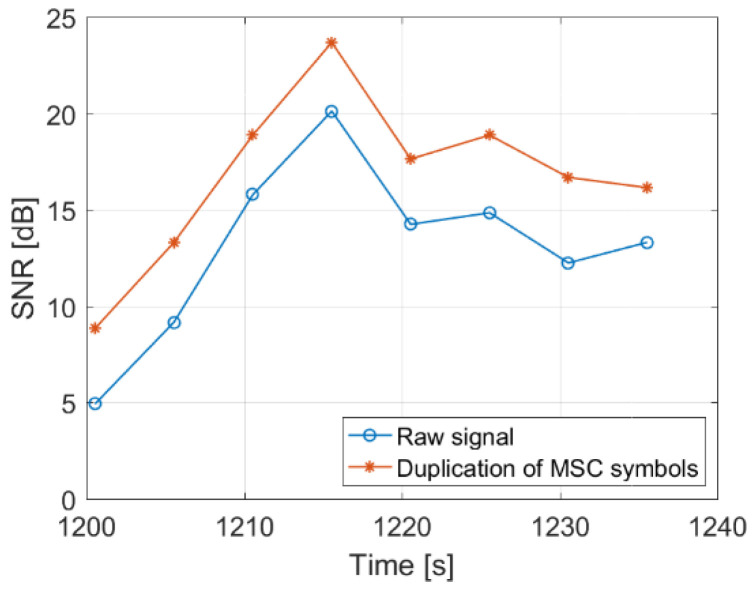
Improvement of SNR achieved by MSC symbols duplication.

To evaluate the performance of this method, the Signal to Noise Ratio (SNR) was estimated for the object detected in the processed signal that was shown in Figure 19. The noise level was averaged over the bistatic velocity/range plane (excluding the clutter zone at *v* ≈ 0), and the signal level was a peak value of the target area bounded with the red rectangles in Figure 19. The comparison of SNR values obtained this way with the raw signals (i.e., without any modifications) and with the method described in Section 3.3 is depicted in Figure 22, showing an average gain of 3.6 dB.

An equally good improvement in the detection plot can be obtained by copying just one MSC symbol from the previous DAB frame in place of the Null symbol, leaving all the peaks in the signal’s autocorrelation function, as depicted in Figure 12. It turns out that the presence of repeatable intervals without the emission of the signal (i.e., the Null symbols) is the reason for periodical artifacts observed in the bistatic velocity/range plane in case of long integration times that cover multiple DAB frames. After fixing these short discontinuities in the received signal (in a way that does not introduce additional noise to the signal), the periodical artifacts are no longer visible in the detection results, as depicted in Figure 18, Figure 19 and Figure 20.

### Computational Complexity Analysis

The computational costs of the described solution result mainly from calculating the moving averages (2) and (3) of the complex signal’s magnitude. In practical implementation, these computations may be simplified by calculating and comparing squared magnitudes without square root calculations

(10) $$p\left(n\right)=\frac{1}{N}\overset{N-1}{\underset{k=0}{\sum }}s\left(n-k\right),$$

(11) $$p_{\mathrm{avg}}\left(n\right)=\frac{1}{M}\overset{M-1}{\underset{k=0}{\sum }}s\left(n-k\right)$$

where *s*(*n*) is a squared magnitude of the signal’s complex sample

(12) $$s\left(n\right)=x_{\mathrm{real}}{\left(n\right)}^{2}+x_{\mathrm{imag}}{\left(n\right)}^{2}.$$

The moving average filters (10) and (11) can be implemented recursively by adding the current value of (12) to the previously calculated $p\left(n-1\right),\;\;p_{avg}\left(n-1\right)$, and subtracting its historical value (from the beginning of the time window), fetched from a circular buffer. Moreover, the sum of squares (12) can be calculated only once for each signal sample, then used in both moving averages and stored in the buffer (with the length of *M*). An additional real multiplication may be performed for each sample to perform thresholding by comparing (10) with the scaled value of (11). After the proposed optimization, the calculation of (10) and (11), and thresholding for each sample would require only three real multiplications, five additions/subtractions, and two divisions, as summarized in Table 2. Furthermore, the divisions may be cheaply realized by bit shifting, provided that *N* and *M* are the powers of two. It should be kept in mind that the results of computations would be valid after filling up the circular buffer (i.e., after processing *M* input samples) and should be ignored prior to that point.

## 5. Conclusions

The proposed modification of the received DAB signals at the transmission-frame level has very little computational complexity since it employs only two moving-average computations, thresholding and synchronous copying of parts of the two input signals. Thus, it may be introduced at the beginning of the signal processing pipeline designed for the DAB-illuminated passive bistatic radar, as shown in Figure 13, with a minimal computational cost. Such an improvement allows extended integration time (i.e., much longer than the DAB transmission frame duration). This, in turn, provides increased Doppler resolution (desired in the presence of a broadband sea-clutter) without periodical artifacts in range/Doppler plots caused by the time structure of the DAB signal, and with around 3 dB of gain in SNR, when compared to the processing of raw DAB signals.

## Figures and Tables

**Table 1:** Main parameters of the DAB signal in Mode I.

Parameter	Value
Number of transmitted subcarriers	1536
Subcarrier spacing	1 kHz
Transmission frame duration ($T_{F}$)	96 ms
Symbol duration ^1^	1246 μs
Null symbol duration	1297 μs
OFDM symbols in frame: ^2^	76
Symbols with PR signal	1
Symbols with FIC data	3
Symbols with MSC data	72

^1^ With guard interval. ^2^ Without Null symbol.

**Table 2:** Computation costs (per sample) of the proposed algorithm.

Operation	Number of Operations
Real multiplications	3
Real divisions ^1^	2
Real additions	3
Real subtractions	2

^1^ May be replaced by bit shifting for certain values of *M*, *N*.

## Data Availability

Not applicable.

## Supplementary Materials

The following supporting information can be downloaded at: https://www.mdpi.com/article/10.3390/s22010378/s1, Videos S1 and S2: Subsequent PCL detection results without modifications in DAB signal, Figures S3 and S4: Subsequent PCL detection results with MSC symbols copied in place of Null, PR, FIC symbols in DAB frame.
