# Supplementary figures and images for: DAB Signal Preprocessing for Passive Coherent Location
Source: Sensors (Basel). 2022 Jan 5;22(1):378. doi: 10.3390/s22010378 (PMC8749620; doi:10.3390/s22010378)

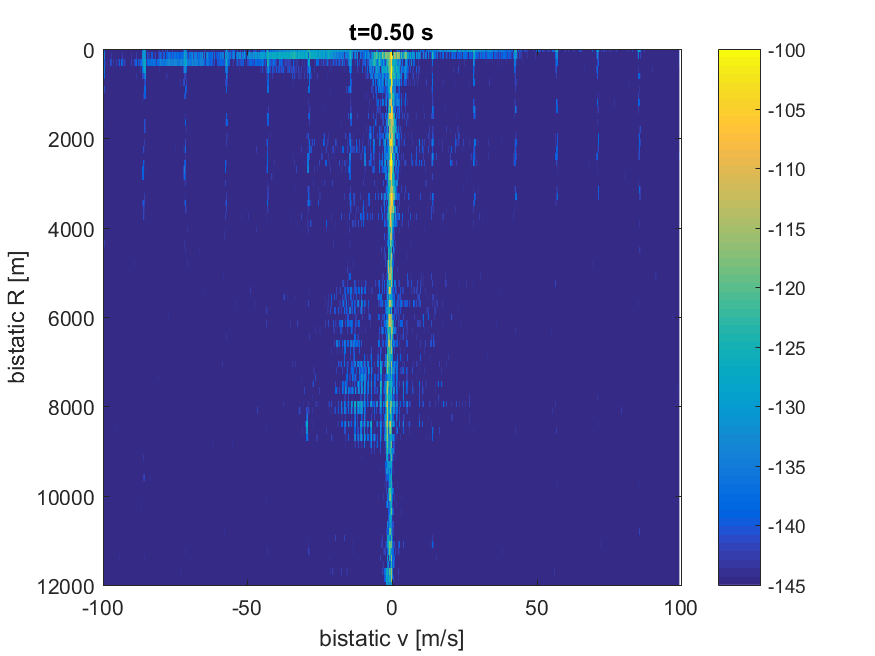

Supplement: Supplementary file 1 [file sensors-22-00378-s001.zip › s3.gif]

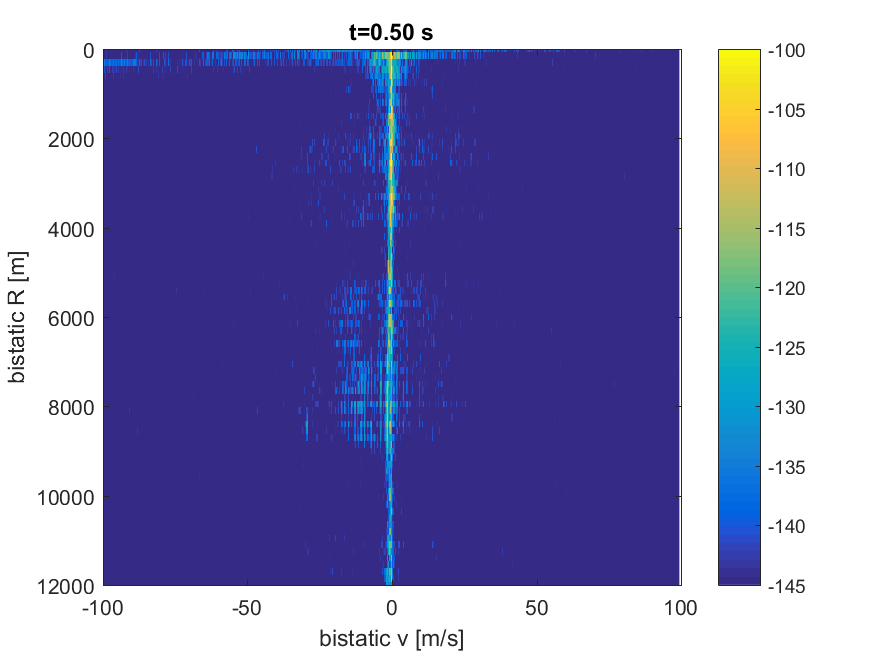

Supplement: Supplementary file 1 [file sensors-22-00378-s001.zip › s4.gif]
